# Splenic Involvement in Hereditary Hemorrhagic Telangiectasia

**DOI:** 10.1155/2016/3212947

**Published:** 2016-10-11

**Authors:** Susumu Takamatsu, Kota Sato, Shunsuke Kato, Hiroto Nagano, Shunro Ohtsukasa, Yasuyuki Kawachi

**Affiliations:** Department of Surgery, Musashino Red Cross Hospital, 1-26-1 Kyonan-cho, Musashino-shi, Tokyo 180-8610, Japan

## Abstract

A 33-year-old man who presented with prolonged epigastric pain was referred to our hospital. He had experienced recurrent epistaxis and had a family history of hereditary hemorrhagic telangiectasia. Computed tomography and magnetic resonance imaging revealed splenomegaly and a 9 cm hypervascular mass in his spleen. Computed tomography also showed a pulmonary arteriovenous malformation and heterogeneous enhancement of the liver parenchyma, suggesting the presence of arteriosystemic shunts and telangiectases. Based on these findings, the patient was definitely diagnosed with hereditary hemorrhagic telangiectasia according to Curaçao criteria. He underwent splenectomy, and his symptoms disappeared after surgery. Pathological examination of the resected specimen revealed that the hypervascular lesion of the spleen was not a tumor but was composed of abnormal vessels associated with hereditary hemorrhagic telangiectasia. Symptomatic splenic involvement may be a rare manifestation of hereditary hemorrhagic telangiectasia but can be revealed by imaging modalities.

## 1. Introduction

Hereditary hemorrhagic telangiectasia (HHT; Rendu-Osler-Weber syndrome) is an autosomal dominantly inherited disorder with a prevalence of 1 in 10000 to 1 in 5000 individuals [1]. HHT is characterized by the presence of vascular abnormalities, such as telangiectases and/or arteriovenous malformations (AVMs), in the lungs, brain, gastrointestinal tract, liver, and spinal cord [2]. However, the spleen is rarely affected.

This report described a patient with HHT, who underwent splenectomy for a hypervascular lesion of the spleen; pathologic examination of the resected specimen confirmed splenic involvement in HHT.

## 2. Case Presentation

**Figure 1:**
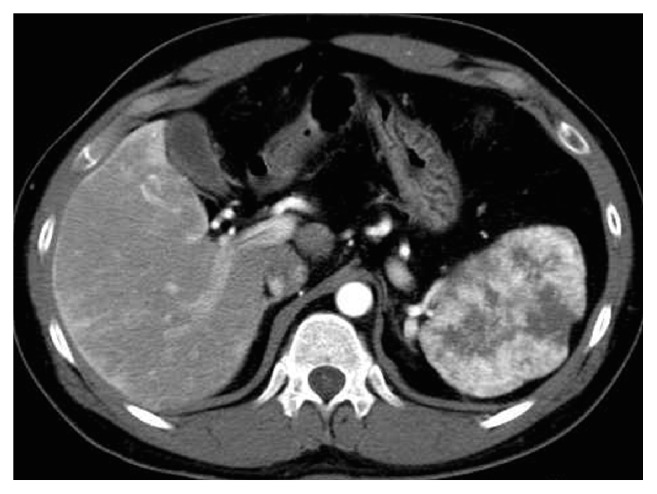
Arterial phase of enhanced CT, showing a 9 cm hypervascular mass in the enlarged spleen of the patient.

**Figure 2:**
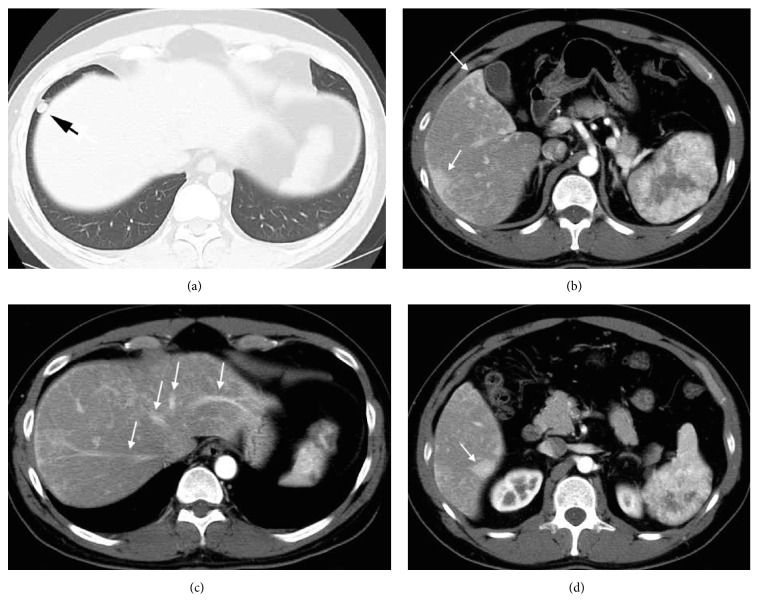
Arterial phase of enhanced CT, showing (a) a pulmonary arteriovenous malformation (arrow); (b) transient perfusion abnormalities (arrow) in the periphery of the liver; (c) three major hepatic veins (arrow), suggesting arteriosystemic shunts of the liver; (d) a large confluent vascular mass (arrow) in the periphery of the liver.

A 33-year-old man who presented with prolonged epigastric pain was referred to our hospital. Outpatient laboratory data were within normal limits, except that his serum carbohydrate antigen 19-9 concentration was slightly elevated, to 79.8 U/mL (normal range 0–36.99 U/mL). Physical examination showed no abnormal findings in the abdomen and no mucocutaneous telangiectases. Based on a personal history of recurrent spontaneous epistaxis and pulmonary AVM and a positive family history, in that his mother has been diagnosed with HHT, this patient had been diagnosed with HHT according to Curaçao criteria [3]. Abdominal enhanced computed tomography (CT) showed a 9 cm hypervascular mass in his enlarged spleen (Figure 1). CT also demonstrated a pulmonary AVM and both transient perfusion abnormalities and a large confluent vascular mass [4] of the liver (Figure 2). Furthermore, arterial phase of enhanced CT showed three major hepatic veins. Both CT and enhanced magnetic resonance imaging (MRI) strongly suggested that the hypervascular splenic lesion was a tumor. Brain MRI, however, showed no evidence of cerebrovascular malformations.

**Figure 3:**
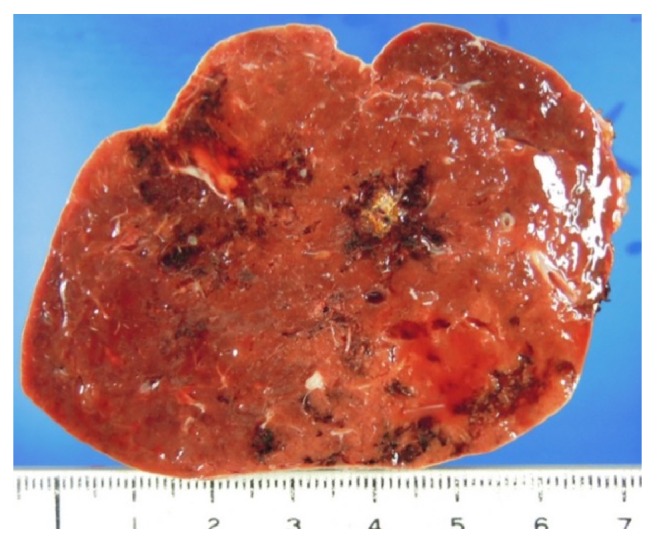
Cut surface of the resected specimen, showing some dark brown hemorrhagic spots, but no evidence of a tumor.

**Figure 4:**
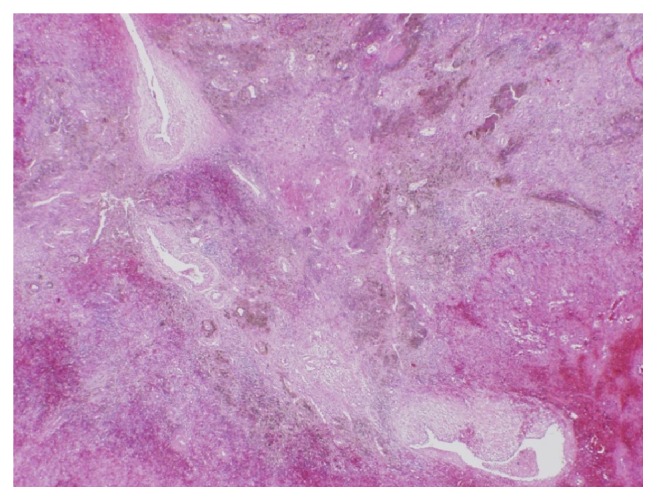
Microscopic findings, showing that the vessels in the dark brown spots were telangiectatic and thin walled, with intimal hyperplasia (hematoxylin and eosin staining, ×20).

The patient underwent splenectomy based on a diagnosis of a symptomatic hypervascular tumor of the spleen. Macroscopic examination of the resected specimen, however, revealed no evidence of a splenic tumor (Figure 3). Rather, pathological examination showed the presence of hemorrhage and hemosiderosis in the area thought to be a hypervascular tumor on CT and MRI. Moreover, the vessels in that area appeared telangiectatic and thin walled, with intimal hyperplasia (Figure 4). These results suggested that the hypervascular lesion in this patient was splenic involvement in HHT. The postoperative course of this patient was uneventful, and he was discharged from the hospital on postoperative day 5. After surgery, his abdominal pain disappeared and he has remained asymptomatic. Genetic testing was not performed.

## 3. Discussion

HHT, also known as Rendu-Osler-Weber syndrome, is a relatively uncommon, autosomal dominant inherited disorder [1]. The abnormal vascular structures in HHT were found to result from mutations in the transforming growth factor-ß/bone morphogenetic protein signaling pathway genes endoglin [5, 6] and activin type-II-like receptor kinase 1 [7]. Clinically, the organs most frequently affected by HHT are the lungs, brain, liver, and gastrointestinal tract [2]. Splenic involvement is considered a rare manifestation, with only a few case reports describing splenic involvement in patients with HHT [8–10].

Although several studies have described the CT findings associated with hepatic involvement in HHT [4, 11], this involvement is not well understood. Retrospectively, CT demonstrated hepatic involvement in the present patient but was unable to show hepatic involvement preoperatively. Moreover, preoperative CT was unable to diagnose the hypervascular lesion of the spleen as splenic involvement of HHT. To our knowledge, no studies to date have fully described splenic characteristics on imaging modalities in patients with HHT, making it difficult to associate specific findings of the spleen with splenic involvement in HHT.

Most previously described HHT patients with visceral involvement have been asymptomatic [3]. Because splenic involvement in this patient was symptomatic, a surgical procedure was deemed necessary. A more precise preoperative diagnosis may have allowed laparoscopic splenectomy, which is regarded as safer and more preferable. The findings in this patient suggest the need to suspect splenic involvement in patients with HHT and to determine imaging characteristics diagnostic of this involvement.

In conclusion, HHT is a relatively uncommon disease, with splenic involvement being an especially rare manifestation. Splenic involvement in HHT should be included in the differential diagnoses of patients with hypervascular lesions of the spleen.
